# Evaluation of waist-to-calf ratio as a diagnostic tool for sarcopenic obesity: a cross-sectional study from a geriatric outpatient clinic

**DOI:** 10.1007/s41999-024-01024-8

**Published:** 2024-08-05

**Authors:** Merve Güner, Yelda Öztürk, Serdar Ceylan, Arzu Okyar Baş, Meltem Koca, Cafer Balci, Burcu Balam Doğu, Mustafa Cankurtaran, Meltem Gülhan Halil

**Affiliations:** 1 Ministry of Health of Republic of Türkiye, Erzurum City Hospital, Erzurum, Turkey; 2 Ministry of Health of Republic of Türkiye, Eskişehir City Hospital, Eskişehir, Turkey; 3 Ministry of Health of Republic of Türkiye, Antalya City Hospital, Antalya, Turkey; 4 Division of Geriatric Medicine, Faculty of Medicine, Hacettepe University, Antalya, Turkey; 5 Ministry of Health of Republic of Türkiye, Ankara Etlik City Hospital, Ankara, Turkey

**Keywords:** Calf circumference, Sarcopenic obesity, Waist circumference, Waist-to-calf ratio

## Abstract

**Background:**

Proponents argue that a high waist-to-calf ratio (WCR) may indicate an imbalance between muscle and fat in the body, making it a potential predictor for sarcopenic obesity (SO). The WCR is a new index incorporating both measurements, providing a reliable approach for assessing the imbalance between abdominal fat and leg muscle mass. The present study aimed to examine the association of WCR with SO and reveal the predictive effect of SO in community-dwelling older adults.

**Methods:**

The study population was composed of 234 geriatric outpatients with obesity. WCR was calculated by dividing the waist circumference (in cm) by the calf circumference (in cm). SO was defined according to the ESPEN and EASO Consensus Statement.

**Results:**

The mean age was 72.7 ± 5.8 years, and 78.7% (n = 175) were female. Eighty-one patients (34.6%) were considered as sarcopenic obese. The WCR was 3.04 [Interquartile range (IQR), 2.88–3.32] in the sarcopenic obese group, and in the nonsarcopenic obese group, it was 2.82 [IQR, 2.7–3.0] (p < 0.001). Independent of age, sex, nutritional and frailty status WCR was associated with SO (OR 12.7, 95% CI 4.0–40.1 and p < 0.001). The cut-off value of WCR for SO was calculated as 2.94 with 72.8% sensitivity and 67.3% specificity (Area Under Curve: 0.72 and Positive likelihood ratio: 2.23, p < 0.001).

**Conclusions:**

WCR, a simple and accessible method, indicates promise as a possible and potential diagnostic tool for SO in community-dwelling older adults.

## Key summary points

**Aim:**

Sarcopenic Obesity (SO) is a complex health concern requiring effective predictors for early detection and intervention. The Waist-to-Calf Ratio (WCR) is a new index that incorporates both measurements, providing a promising approach for assessing the imbalance between abdominal fat and leg muscle mass. This study assessed the association of the WCR with SO in community-dwelling older adults. the imbalance between abdominal fat and leg muscle mass. The present study aimed to examine the association of WCR with SO and reveal the predictive effect of SO in community-dwelling older adults.

**Findings:**

Our findings reveal a significant association between WCR and SO, independent of age, sex, malnutrition, and frailty.

**Message:**

The primary discovery of this study is that WCR may be a potential valuable predictor for SO in community-dwelling older adults.

## Introduction

Sarcopenic obesity (SO) is a syndrome that results in increased body weight and reduced muscle mass/strength. Since the alteration in body composition occurred with the aging and increased prevalence of obesity in the geriatric population, SO is a common and yet overlooked health problem [1, 2]. Recent studies have indicated that SO had a worse cardiovascular prognosis than those with either sarcopenia and obesity alone [3]. Obesity and sarcopenia in older individuals may accelerate physical impairment, morbidity, and mortality [4]. Older adults who are obese and have low muscle mass might experience the combined negative effects of both conditions, leading to a higher risk of physical impairment [5].

Different definitions have been proposed for the diagnosis of both obesity and sarcopenia in recent years. The new diagnostic algorithm was suggested in 2022 by The European Society for Clinical Nutrition and Metabolism (ESPEN) for detecting SO [6]. According to this algorithm screening, diagnosis, and staging were supported to be assessed. For the diagnosis skeletal muscle strength and mass should be evaluated. Different studies have described obesity by BMI (kg/m2), fat mass, waist circumference (WC), and visceral fat area using different cutoff thresholds. For the recognition of sarcopenia, especially decreased muscle mass, several methods could be preferred including dual-energy x-ray absorptiometry (DXA), bioelectrical impedance analysis (BIA), anthropometry, creatine dilution test, ultrasonography (US), computed tomography (CT), and magnetic resonance imaging (MRI) [7]. However, all these methods had some limitations. For DXA, these factors include cost, and variability in tissue thickness, as a result, reduce accuracy in individuals with obesity and lead to discrepancies in body composition and functional parameters [8].

Furthermore, obesity affects the body’s hydration status, which reduces the accuracy of BIA [9]. CT and MRI are difficult to access, high-cost, and rarely used only for the detection of muscle mass. Therefore, there is a need for inexpensive, simple anthropometric methods that can be evaluated during a physical examination.

The waist-to-calf ratio (WCR) is a relatively new concept in the field of obesity research. It is calculated by dividing WC by calf circumference (CC) and is believed to offer insights into body composition and distribution of fat and muscle. In previous studies, it has been shown that WCR is useful for assessing abdominal obesity and related health risks, including metabolic conditions like insulin resistance and cardiovascular diseases such as carotid atherosclerosis in diabetic patients [10]. Additionally, high WCR is linked to cognitive decline in older adults and increased frailty, highlighting its importance in evaluating overall health [11]. Proponents argue that a high WCR may indicate an imbalance between muscle and fat in the body [12], making it a potential predictor for SO. The WCR is a new index incorporating both measurements, providing a potential approach for assessing the imbalance between abdominal fat and leg muscle mass. Therefore, it is beneficial to utilize WCR for the reasons stated above for identifying SO.

The present study aimed to examine the association of simple anthropometric measurement, WCR, with SO and explore to use as a diagnostic tool for SO in community-dwelling older adults.

## Methods

### Study population and study design

This cross-sectional study enrolled 234 participants aged 65 whose BMI ≥ 30 kg/m^2^ were admitted to the geriatric outpatient clinic between October 2023 and April 2024. Written and verbal informed consent were obtained from all participants and/or their legal guardians. Patients with malignancy, chronic kidney disease, and acute illness, patients who have undergone major surgery within the past six months, physical disabilities that impair the ability to perform required measurements for the study protocol, patients with edema in their extremities or any ambulation, and patients who are unwilling to participate in the current study were excluded. The study was approved by the Ethics Review Board of Hacettepe University (Decision number:2023/09–40 and Research number: SBA 23/225), and conducted following the Declaration of Helsinki.

Demographic features including age, sex, educational level, anthropometric parameters, and living conditions were recorded. Chronic diseases (such as diabetes mellitus, hypertension, coronary artery disease, hyperlipidemia, chronic heart failure, and hypothyroidism) and geriatric syndromes (such as frailty, malnutrition, osteoporosis, dementia, depression, urinary incontinence, fall, and polypharmacy) were defined by comprehensive geriatric assessment, laboratory tests, participants’ self-reports, and a review of current medications. Multimorbidity is accepted as the presence of two or more chronic conditions at the same time.

### Comprehensive geriatric assessment

Basic activities of daily living (ADL) [13, 14] (0–6 Points) and instrumental activities of daily living (IADL) (0–8 points) [15] are validated scales used to measure the independence of patients and functional ability. Basic ADLs consist of six activities these are bathing, dressing, toileting, continence, transferring, and feeding. Using the phone, shopping, food preparation, housekeeping, laundry, transporting, taking medications, and handling finances are the components of IADL. The Mini-Nutritional Assessment Short Form (MNA-SF) (0–14 points) was used to determine nutritional status, and scores of less than 11 were considered malnutrition and malnutrition risk [16]. The frailty status of the patients was defined via the Clinical Frailty Score (CFS) (1–9 points) [17–19]. According to CFS, patients who were level 4 and more were accepted as living with frailty. Geriatric syndromes (including osteoporosis, dementia, depression, urinary incontinence, falls, and polypharmacy) defined by comprehensive geriatric assessment were also recorded.

Muscle strength was assessed by Handgrip Strength (HGS) and measured using a calibrated hand-held dynamometer (T.K.K.5401; Takei III Smedley Type Digital Dynamometer Takei Scientific Instruments, Tokyo, Japan). Measurements were taken while the participants were standing with their arms positioned parallel to the floor. The highest of the 3 repeated measurements was used in the analysis. HGS < 16 and < 27 kg, for women and men, respectively, were taken as cutoff values to assess muscle strength [7]. Low physical performance was defined as gait speed ≤ 0.8 m/s during a 4 m walking test using a manual stopwatch, in terms of its convenience to use and ability to predict sarcopenia-related outcomes [7]. Gait speed was calculated as the average of two measurements.

### Anthropometric measurements

Weight and height were measured using standard procedures with participants wearing light clothing without shoes. BMI was calculated by dividing body weight in kg by height in meters squared (kg/m2). WC was measured by a tape measure on the level of the umbilicus at rest to avoid the impact of breathing; hip circumference (HC) was measured on a level parallel to the floor at the largest circumference of the buttocks; mid-upper arm circumference (MAC) was measured from the midpoint between the acromial and olecranon protrusions in an upright, standing position while the arm was twisted by 90°from the elbow; and CC was measured from the widest part of the legs by pressing the feet onto hard and plain ground [20–22]. WCR was calculated by dividing the WC measurement (in cm) by the CC measurement (in cm).

### Assessment of sarcopenic obesity

BIA was performed by Bodystat QuadScan 4000 (BodyStat Ltd., Douglas, Isle of Man, British Isles) to evaluate muscle mass while patients in a supine position on the bed with abduction of extremities to avoid contact with each other and the trunk after overnight fasting with empty bladder. Body compositions and muscle masses were evaluated by measuring the fat-free mass index (FFMI). FFMI (kg/m2) is calculated by dividing the fat-free mass (FFM) by the square of height. FFMI thresholds of 15 kg/m^2^ for women and 17 kg/m^2^ for men were used to define low muscle mass [23, 24].

Diagnosis of SO was based on the following parameters:

- Obesity was defined by a high BMI (≥ 30 kg/m.^2^) [25]
- Sarcopenia, diagnosed by low muscle strength and defined by low HGS (< 27 kg for males and < 16 kg for females) and BIA (Fat mass % > 31% for male, and > 43.0% for female; or Skeletal Muscle Mass/Weight ≤ 37% for male, and ≤ 27.6 for female [6].

This is the current definition and the diagnostic criteria of SO recommended by the ESPEN and EASO [6]. Afterward, participants were classified into 2 groups: Patients with both altered skeletal muscle functional parameters and altered body composition were composed of sarcopenic obese group, and rest of the study population was accepted as non-sarcopenic obese group.

### Statistical analysis

The statistical analyses were performed using the SPSS software, version 26. The variables were assessed using visual (histograms, probability plots) and analytical (Kolmogorov-Simirnow test) methods to determine whether or not they were normally distributed. Descriptive analyses were shown using percentages for categorical variables, means and standard deviations for normally distributed variables, and medians and [interquartile range (IQR)] for non-normally distributed and ordinal variables. The chi-square or Fisher exact test was used to compare differences between the categorical variables as appropriate. The Mann Whitney U and Student’s t-tests were used to compare non-normally and normally distributed variables, respectively. P < 0.05 was considered statistically significant. The estimation capacity of WCR to predict SO was analyzed using a receiver operating characteristics (ROC) curve analysis. The sensitivity, specificity, and positive and negative predictive values were presented when a significant cutoff value was present with 95% confidence interval (CI) and a 5% level of significance (p < 0.05). The associations of WCR with SO were determined using univariable and multivariable logistic regression analyses with odds ratio (OR) and 95% CI. The adjusted model included age, sex, CFS (score), nutritional status (MNA-SF < 12 points), multimorbidity, and WCR. Hosmer–Lemeshow test (p > 0.05) was applied to assess model fit.

## Results

Among 234 participants, the mean age was 72.7 ± 5.9 years, and 74.8% (n = 175) were female. Among the whole study group, 81 patients (34.6%) were considered sarcopenic obese. The female ratio was 63.0% in sarcopenic obese group and in nonsarcopenic obese group it was 81.0%, and the difference was significant (p = 0.004). The patients with SO were substantially older than nonsarcopenic obese participants (the mean age 74.9 ± 6.9 years and 71.5 ± 4.9 years, respectively and p < 0.001). No differences were observed in other demographic features of the two groups. Table 1 presents the main sociodemographic and clinical characteristics of the participants.

**Table 1:** The baseline characteristics of the study population

	Sarcopenic obese (n = 81)	Non-sarcopenic obese (n = 153)	p
Age, years	74.9 ± 6.9	71.5 ± 4.9	< 0.001
Sex, female	51 (63.0)	124 (81.0)	0.004
Education, < 5 years	57 (72.2)	103 (69.1)	0.64
Marital status, married	63 (79.8)	122 (81.4)	0.58
Living alone	12 (15.2)	23 (15.3)	0.98
BMI, kg/m^2^	34.7 [31.9–40.2]	34.3 [31.6–36.3]	0.48
Handgrip strength, kg	14.8 [13.0–21.8]	20.7 [17.8–25.0]	< 0.001
Female	13.6 [11.5–14.9]	20.1 [17.0–22.0]	< 0.001
Male	22.8 [18.3–24.3]	32.6 [30.0–35.4]	< 0.001
Mid-upper arm circumference, cm	31.0 [29.0–34.0]	32.0 [29.8- 34.0]	0.12
Hip circumference, cm	115.0 [108.0–125.0]	113.0 [109.0–119.0]	0.11
Calf circumference, cm	37.0 [34.0–39.0]	39.0 [36.0–40.0]	0.001
Waist circumference, cm	114.0 [105.0–123.0]	108.0 [103.5–115.0]	0.004
Waist-to-calf ratio	3.05 [2.87–3.32]	2.82 [2.70–3.00]	< 0.001
Chronic conditions			
Hypertension	72 (88.9)	119 (77.8)	0.037
Diabetes mellitus	52 (64.2)	81 (52.9)	0.098
Hyperlipidemia	21 (25.9)	31 (20.3)	0.32
Chronic heart failure	10 (12.3)	8 (5.2)	0.052
Coronary artery disease	21 (25.9)	32 (20.9)	0.38
Multimorbidity (≥ 2 disease)	77 (95.1)	131 (85.6)	0.029
Polypharmacy	59 (72.8)	95 (62.1)	0.099
Comprehensive geriatric assessment			
Basic ADL	6.0 [5.0–6.0]	6.0 [5.0–6.0]	0.021
Instrumental ADL	7.5 [5.5–8.0]	8.0 [8.0–8.0]	< 0.001
Frailty, CFS	54 (66.7)	56 (36.6)	< 0.001
CFS, score	4.0 [3.0–5.0]	3.0 [3.0–4.0]	< 0.001
Malnutrition and malnutrition risk (MNA-SF < 12 points)	23 (29.9)	23 (15.5)	0.011
4- meter walking, sec	1.6 [0.7–5.5]	1.4 [0.9–4.1]	0.025
Timed-up and go, sec	11.4 [7.9–13.2]	11.4 [9.6–17.3]	0.16
Osteoporosis	19 (23.5)	38 (24.8)	0.82
Incontinence	32 (39.5)	57 (37.3)	0.74
Falls	20 (24.7)	28(18.3)	0.48
Depression	12 (14.8)	18(11.8)	0.51
Dementia	15 (18.5)	9 (5.8)	0.002

*BMI* body-mass index; *ADL* activities of daily living; *CFS* clinical frailty scale; *MNA-SF* mini-nutritional assessment- short form. Variables were presented as number (%), mean ± SD, or median [IQR]

Anthropometric parameters regarding CC was lower and WC was significantly higher in the sarcopenic obese group than in the nonsarcopenic obese group (p = 0.001 and p = 0.004, respectively), however, there were no differences found between the two groups, including BMI, HC, and MAC. The WCR was 3.05 [2.87–3.32] in the sarcopenic obese group and in the nonsarcopenic obese group, it was 2.82 [2.70–3.00]. The WCR was significantly higher in patients with SO than in patients without SO (p < 0.001) (Table 1).

The prevalence of hypertension was higher in patients with SO than in patients without SO (88.9% vs.77.8%, p = 0.037). However, other chronic conditions were similar in the two groups (p > 0.05 for all). Furthermore, multimorbidity was more common in patients with SO (p = 0.029), on the other hand, no difference was found between the two groups according to polypharmacy (p > 0.05).

Basic and instrumental ADL scores were lower in the sarcopenic obese group than in the nonsarcopenic obese group. The frailty defined by CFS was observed more commonly in patients with probable SO and the difference was statistically significant (66.7% vs. 36.6%, respectively, and p < 0.001). Risk of malnutrition and malnutrition, and dementia were more common in patients with SO than in patients without SO (p < 0.05 for all). No differences were observed in other geriatric syndromes regarding incontinence, fall history, osteoporosis, and depression (p > 0.05 for all).

**Fig. 1:**
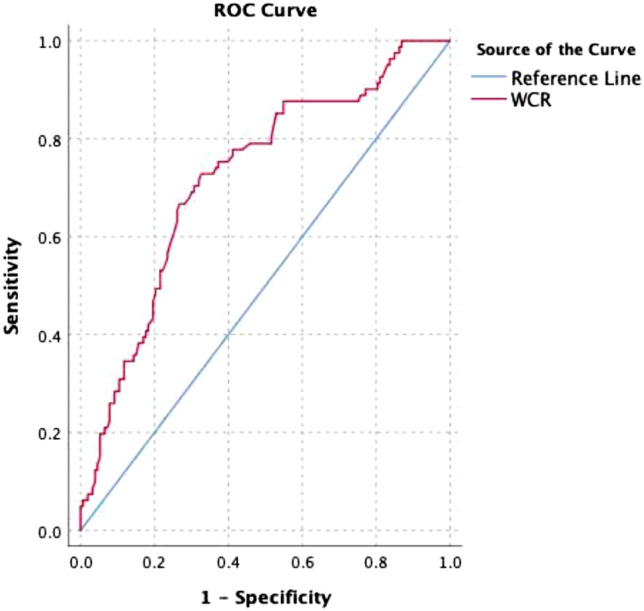
Receiver operating characteristic (ROC) curves of waist-to-calf ratio (WCR) (red) and reference line (blue) obtained when predicting sarcopenic obesity. Area under the ROC curve was 0.72 for the WCR

Table 2 shows the ROC analysis for WCR to predict SO. The cut-off value of WCR for the prediction was calculated and it was found 2.94 with 72.8% sensitivity and 67.3% specificity. The positive and negative likelihood ratios were calculated as 2.23 and 0.40, respectively. The ROC analysis curve is shown in Fig. 1.

**Table 2:** Receiving operator curve of the waist-to-calf ratio to predict sarcopenic obesity

	Cut-off	AUC	95% CI	Sensitivity	Specificity	p
WCR	2.94	0.72	0.65–0.79	72.8	67.3	< 0.001

*WCR* waist-to-calf ratio; *AUC* area under curve; *CI* Confidence Interval

We assessed the link between WCR and SO (Table 3). WCR was significantly associated with SO in the unadjusted model (OR 13.3, 95% CI 4.9–35.7 and p < 0.001). Furthermore, independent of age, sex, multimorbidity, nutritional, and frailty status WCR was associated with SO (OR 12.7, 95% CI 4.0–40.1 and p < 0.001).

**Table 3:** Multivariable binary logistic regression analysis of sarcopenic obesity

	OR	95% CI	p
Unadjusted model			
WCR	13.3	4.9–35.7	< 0.001
Adjusted model			
Age, years	1.0	1.0–1.1	0.18
Sex, female	4.2	1.9–9.2	< 0.001
CFS	2.4	1.5–3.7	< 0.001
MNA-SF, score	1.1	0.4–2.7	0.85
Multimorbidity	2.5	0.7–8.8	0.16
WCR	12.7	4.0–40.1	< 0.001

*WCR* waist-to-calf ratio; *CFS* clinical Frailty Scale. The adjusted model included age, sex, Clinical Frailty Scale (score), malnutrition, multimorbidity, and waist-to-calf ratio

## Discussion

SO is a complex and emerging health issue characterized by the simultaneous presence of low muscle mass and excess body fat in individuals, often leading to various health complications. Identifying reliable predictors for SO is crucial for early detection and effective intervention. In this study, we would like to explore the merits and limitations of the WCR as a screening tool for SO and consider its clinical implications. We found that WCR is significantly associated with SO regardless of age, sex, malnutrition, and frailty. The foremost finding of the present study is that WCR could predict SO in community-dwelling older adults.

WCR can complement existing tools for assessing obesity and muscle health, such as BMI and waist-to-hip ratio (WHR). Previous studies recommended that anthropometric proportions of abdominal obesity, such as waist-hip ratio (WHR) and WC, are preferred indicators of CVD risk over BMI [26]. Another study revealed that muscle mass was correlated with BMI, WHR, and some other anthropometric measurements, which may serve as early indicators in the diagnosis of SO [27]. Moreover, Park et al. suggested that along with other anthropometric measurements, WHR cannot appropriately reflect reduced muscle mass in obese patients [28]. Even though WHR and BMI are widely used, they don’t specifically account for muscle mass, meanwhile, CC is another form of lean mass and peripheral subcutaneous fat [10]. SO is a unique condition involving not just fat accumulation but also muscle wasting. Therefore, WCR might provide a more comprehensive view by considering both factors. WCR is relatively straightforward, non-invasive, and suitable for clinical practice, and may complement existing diagnostic tools, contributing to better management of probable SO. However, further research is needed to establish reliable cutoff values and assess their accuracy in predicting SO.

WCR is a valuable metric that can provide insights into an individual's body composition and potential health risks. It is particularly relevant when assessing issues related to abdominal obesity and metabolic conditions, such as insulin resistance. Hence, individuals with SO are at an elevated risk for metabolic syndromes due to the combination of excess fat and reduced muscle mass, which further exacerbates insulin resistance. A study conducted by Rao in 2021 found a significant association between WCR and carotid atherosclerosis in diabetic patients, indicating that an increased WCR may be linked to a higher risk of this cardiovascular condition [10] which might mirror the cardiovascular risks faced by sarcopenic obese individuals.

Furthermore, WCR has also been explored concerning cognitive function, especially among older adults. Research conducted by Cao in 2023 concluded that a high WCR can negatively affect cognitive function in older individuals [11]. This suggests that central obesity, which is often indicated by a high WCR, may contribute to cognitive decline. Additionally, a study involving over 2000 participants, as reported by Dai in 2023, demonstrated a significant association between higher WCR and frailty [12]. Both conditions could be accepted as consequences of inflammation and metabolic dysregulation, which are also key points for SO.

These findings suggest that individuals with a higher WCR, indicative of high central fat and low lean body mass, may be at an increased risk of developing SO further increasing the risk of metabolic and functional impairments.

We acknowledged that the study had some limitations.One of them is that our SO-cut-off value has moderate AUC, sensitivity, and specificity with low positive and negative likelihood ratios. We concur that the WCR, as a standalone measure, may have limited utility in clinical practice for screening SO. However, it can still be considered as part of a multi-faceted approach, potentially combined with other clinical indicators and diagnostic tools to improve overall predictive accuracy. Secondly, body composition could be influenced by numerous factors, including sex, genetics, age, and physical activity levels. Future research should aim to provide a more complete assessment of SO with high AUC, sensitivity, and specificity, including sex-specific cut-off points. There are also some strengths of the present study. The scientific literature on the WCR as a predictor for probable SO is relatively scarce. The WCR could serve as an early warning sign for SO, allowing for timely interventions to prevent its development or progression. More studies are needed to validate its effectiveness and establish its reliability with larger and balanced cohorts analyzing the differences between males and females.

In conclusion, SO is a multifaceted health concern that demands effective predictors for early detection and intervention. While the WCR, a simple and accessible method, shows promise as a potential and possible predictor for SO, further research is needed to establish its validity and clinical utility with sex-specific cut-offs in larger and balanced cohorts. As our understanding of this condition evolves, integrating the WCR into comprehensive assessments may become a valuable tool in managing and preventing SO.

## Data Availability

The data that support the findings of this study are not openly available due to reasons of sensitivity and are available from the corresponding author upon reasonable request.
